# Ischaemic Heart Disease Masquerading as Headache: A Case Series

**DOI:** 10.1002/ccd.31521

**Published:** 2025-03-28

**Authors:** Ibrahim Antoun, Ayman Helal, Nancy Wassef, Mohsin Farooq

**Affiliations:** ^1^ Department of Cardiology Kettering General Hospital Kettering UK; ^2^ Department of Cardiovascular Sciences University of Leicester Leicester UK; ^3^ Department of Cardiology Gloucestershire Hospital Foundation Trust Gloucestershire UK

**Keywords:** angina, coronary angiogram, headache, myocardial infarction, percutaneous coronary intervention

## Abstract

Headache is a rare yet clinically significant presentation of ischaemic heart disease (IHD). While chest pain is the hallmark symptom of myocardial ischaemia (MI), some patients present with atypical symptoms, such as headaches, which lead to diagnostic challenges and potential delays in treatment. This case series highlights the diagnostic complexity and clinical significance of headache‐predominant presentations of both acute and chronic coronary syndromes, emphasizing the need for a comprehensive differential diagnosis in patients with cardiovascular risk factors. We present two cases where headache was the primary symptom of MI. The first case describes an acute ischaemic event wherein the headache preceded the onset of classic cardiac symptoms, leading to the identification of an occluded obtuse marginal artery. This was the second case in our institution where a previous patient presented with exertion‐induced headaches, ultimately diagnosed as a chronic total occlusion of the left anterior descending (LAD) artery, which was successfully revascularised. Two years later, the same patient re‐presented with acute coronary syndrome secondary to disease in a different coronary artery and his presentation was solely with headache. Both cases were successfully managed with percutaneous revascularisation, resulting in the resolution of symptoms and reinforcing the link between headache and CAD. These cases underscore the importance of considering ACS and chronic stable angina in patients presenting with unexplained headaches, particularly when symptoms are exertional or pressure‐like. Early cardiac assessment, including ECG and further imaging when indicated, is essential for timely intervention. Raising the awareness of exertional headache as a potential ischaemic symptom may facilitate earlier diagnosis and prevent adverse outcomes. Further research is required to elucidate the mechanisms underlying headaches in MI and refine diagnostic approaches for atypical cardiac presentations.

## 1 Introduction

Headache is a well‐documented yet often under‐recognized symptom of cardiovascular diseases, particularly within the context of acute coronary syndrome (ACS) and chronic stable angina [1, 2, 3]. While chest pain remains the predominant symptom leading to the diagnosis of myocardial ischaemia (MI), a subset of patients presents with atypical symptoms such as headaches, which may lead to mismanagement. The pathophysiological mechanisms linking headaches with MI are not fully understood, but proposed explanations include autonomic dysfunction, referred pain mechanisms, and vascular endothelial dysfunction [3]. Previous studies have highlighted the association between exertional headaches and significant CAD, particularly in patients without traditional anginal symptoms [3, 4]. This case series presents two patients with headache‐predominant presentations of MI. The first case involves an acute ischaemic event in which headache was the initial symptom, later evolving into classic cardiac chest discomfort. The second case describes exertional headaches as the primary manifestation of chronic total occlusion (CTO) of the left anterior descending artery (LAD), with subsequent recurrence linked to progressive CAD in a different coronary artery.

## 2 Case Presentation

**Figure 1:**
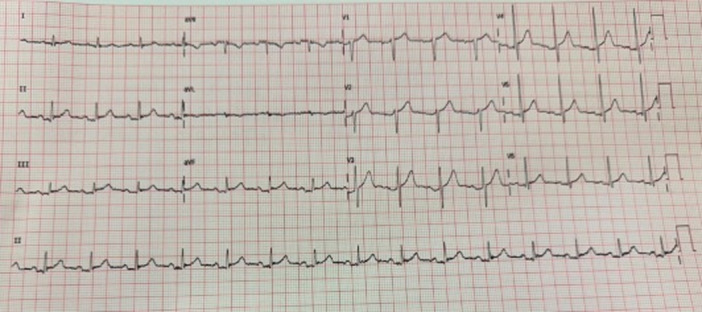
12‐leads electrocardiogram of the patient demonstrating atrial fibrillation with anterolateral ST elevation. [Color figure can be viewed at wileyonlinelibrary.com]

**Figure 2:**
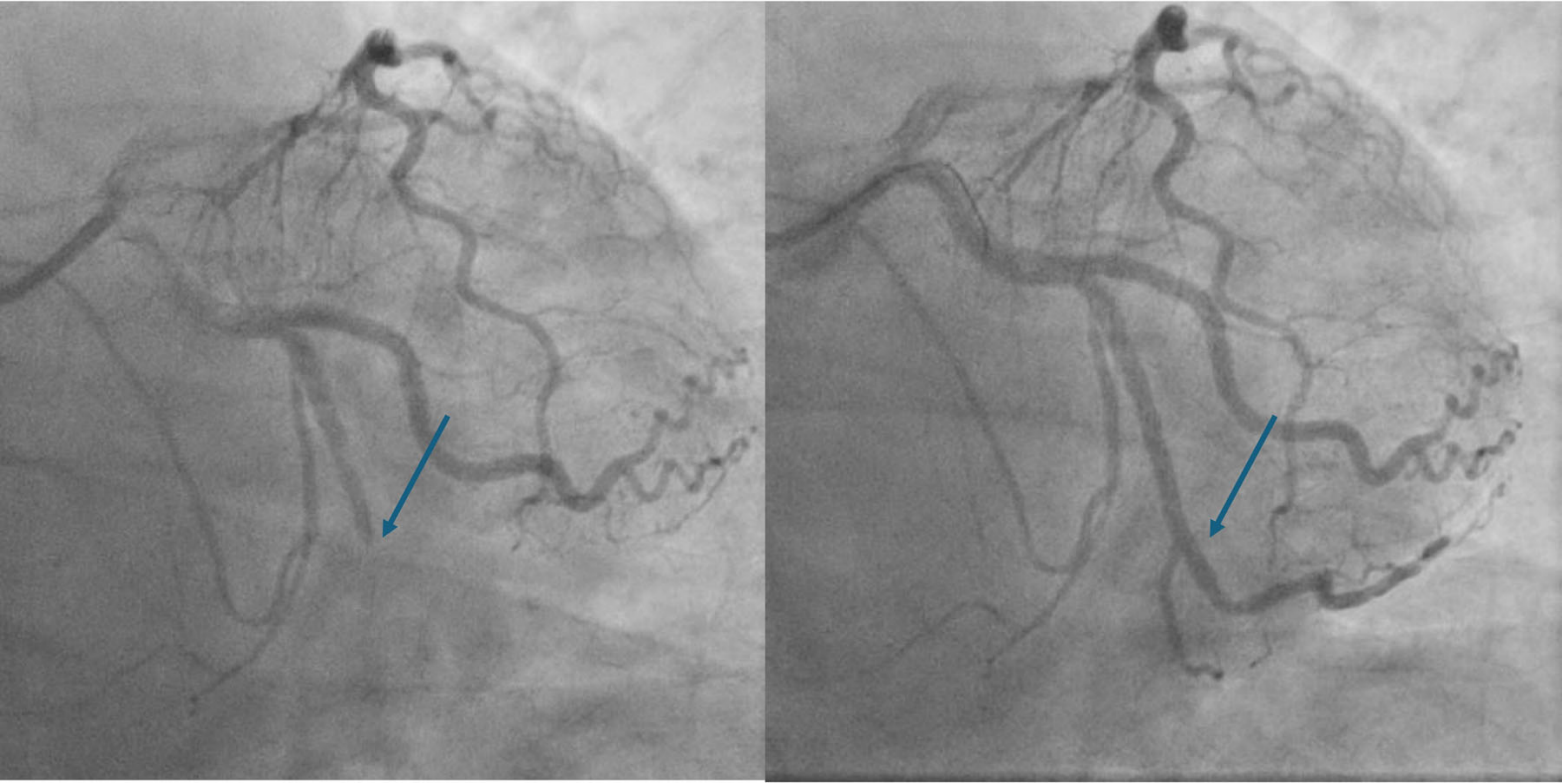
Invasive coronary angiogram demonstrating acutely occluded obtuse marginal artery (left figure), which was successfully stented (right figure). [Color figure can be viewed at wileyonlinelibrary.com]

Our first patient is a 60‐year‐old male who has no past medical history and takes no regular medications. He presented to the emergency department (ED) due to progressively worsening headaches over the preceding 3 h. It was mainly on top of the head and without radiation. There were no other neurological symptoms. Vital signs and physical assessment, including neurological examination, were normal. Routine blood results were normal. Whilst in ED, he started having diaphoresis and central chest tightness. A 12‐lead electrocardiogram (ECG) demonstrated inferior ST elevation (Figure 1). The interventional cardiology consultant on call discussed this with the patient and advised arranging an urgent computed tomography (CT) of the head and an aortogram. This was done to rule out intracranial haemorrhage and aortic dissection. By now, the chest tightness had settled, but the patient continued to have headaches with no resolution of the elevated ST segments. The patient was, therefore, taken to the cardiac catheterization laboratory and underwent emergency coronary angiography, demonstrating an acutely occluded obtuse marginal artery, which was successfully stented (Figure 2). There was rapid and full resolution of the headache post‐stenting. The patient made an uneventful recovery and was discharged home 48 h later without further headaches.

Previously, from our institution, we have presented [5] the case of a younger gentleman in his forties who had presented with severe exertional headaches with mild chest discomfort, which was relieved with rest. These symptoms were triggered solely by exercise. The patient was found to have hyperlipidaemia, and all neurological causes for headache were excluded. He had a non‐diagnostic exercise tolerance test followed by coronary angiography that revealed a CTO of the LAD. The latter was successfully treated with a drug‐eluting stent. Following the procedure, the patient remained free of headache symptoms until 2 years later, when he presented again with ACS. Presenting symptoms were predominantly of headache, and this was similar in character to his previous presentation. He was found to have severe disease in a different coronary artery and underwent a successful angioplasty to his left circumflex artery.

The two patients had complete resolution of headaches following coronary revascularisation. The second patient had a recurrence of the headache only when he presented with another coronary lesion that completely resolved with angioplasty.

## 3 Discussion

Headache is an extremely rare but clinically significant presenting symptom of ACS and chronic stable angina [6, 7, 8]. While chest pain remains the hallmark symptom of ischaemic heart disease (IHD), atypical presentations, including headaches, can delay diagnosis and management. The cases presented highlight two distinct manifestations of IHD: ACS and chronic stable angina. In both cases, headache was the primary or predominant symptom, highlighting the importance of a broad differential diagnosis in patients with cardiovascular risk factors or accompanying symptoms.

The underlying mechanisms linking headache to MI remain incompletely understood. Several hypotheses have been proposed:

1. Autonomic dysfunction: MI triggers autonomic responses, leading to symptoms such as diaphoresis, nausea, and headache. This autonomic response may be mediated by increased sympathetic activation and catecholamine release, which could contribute to vascular headache mechanisms similar to those seen in migraine pathophysiology [9].
2. Referred pain: Cardiac pain can be referred to non‐thoracic regions, including the head, due to shared neural pathways between the heart and upper cervical sensory fibers [7]. This may explain why some patients with MI experience headaches rather than or in addition to chest pain.
3. Endothelial dysfunction and vascular dysregulation: CAD involves endothelial dysfunction and reduced nitric oxide bioavailability, potentially affecting cerebral vasculature and predisposing patients to headaches [10].
4. Exercise‐induced hyper‐perfusion and vasodilation: In patients with fixed coronary stenoses, exertion‐induced ischaemia can lead to systemic hemodynamic changes, potentially affecting cerebral blood flow and leading to exertional headaches [3, 4].

These cases suggest that an ischaemic cardiac aetiology should be considered in patients presenting with unexplained headaches, particularly exertional or pressure‐like headaches, especially in those with cardiovascular risk factors. This aligns with previous reports where headaches, including migraine‐like symptoms, have been associated with silent MI or ACS [1, 2]. Timely recognition and treatment of headache‐predominant ACS and chronic stable angina are crucial for preventing adverse outcomes. Our cases reinforce the importance of early cardiac assessment, including ECG and further imaging or stress testing, to rule out MI. Coronary revascularisation successfully alleviated symptoms in both patients, further supporting the causal relationship between ischaemia and headache.

These cases highlight headaches as an atypical but important presentation of MI. Clinicians should maintain a high index of suspicion for ACS and chronic stable angina in patients with exertional or unexplained headaches, particularly in the presence of cardiovascular risk factors or atypical chest discomfort. Further studies are needed better to characterize the relationship between headaches and IHD, potentially leading to improved diagnostic strategies and earlier intervention for affected patients.

## 4 Conclusion

Our cases highlight the necessity of considering ACS and chronic stable angina in patients who present with atypical symptoms, such as headaches, particularly in those experiencing exertional triggers or possessing cardiovascular risk factors. Atypical ischaemic symptoms may lead to delays in diagnosis and treatment, potentially resulting in adverse outcomes. Early recognition through appropriate diagnostic investigations is vital for timely management.

## Ethics Statement

Informed written consent was obtained from all patients before participating, and their anonymity and confidentiality were strictly maintained. The study adhered to the principles outlined in the Declaration of Helsinki. Given the retrospective nature of this case series and the anonymisation of patient data, formal ethical approval was not required.

## Consent

The authors confirm that written consent was obtained before submission of the case report.

## Conflicts of Interest

The authors declare no conflicts of interest.

## Data Availability

The data that support the findings of this study are available from the corresponding author upon reasonable request.

## References

[1] M. Wang , L. Wang , C. Liu , X. Bian , Z. Dong , and S. Yu , “Cardiac Cephalalgia: One Case With Cortical Hypoperfusion in Headaches and Literature Review,” Journal of Headache and Pain 18 (2017): 24. 28220375 10.1186/s10194-017-0732-3 PMC5318311

[2] A. Bini , A. Evangelista , P. Castellini , et al., “Cardiac Cephalgia,” Journal of Headache and Pain 10 (2009): 3–9. 19139804 10.1007/s10194-008-0087-x PMC3451760

[3] V. González‐Quintanilla , J. Madera , and J. Pascual , “Update on Headaches Associated With Physical Exertion,” Cephalalgia 43, no. 3 (2023): 03331024221146989. 10.1177/03331024221146989 36786294

[4] R. B. Lipton , T. Lowenkopf , Z. H. Bajwa , et al., “Cardiac Cephalgia: A Treatable Form of Exertional Headache,” Neurology 49, no. 3 (1997): 813–816. 9305346 10.1212/wnl.49.3.813

[5] N. Wassef , A. T. Ali , A. Z. Katsanevaki , and S. Nishtar , “Cardiac Cephalgia,” Cardiology Research 5, no. 6 (December, 2014): 195–197, 10.14740/cr361w. 28352454 PMC5358270

[6] J. Auer , R. Berent , E. Lassnig , and B. Eber , “Headache as a Manifestation of Fatal Myocardial Infarction,” Neurological Sciences 22 (2001): 395–397. 11917978 10.1007/s100720100071

[7] A. Ishida , O. Sunagawa , T. ToUMA , Y. Shinzato , N. Kawazoe , and K. Fukiyama , “Headache as a Manifestation of Myocardial Infarction,” Japanese Heart Journal 37, no. 2 (1996): 261–263. 8676553 10.1536/ihj.37.261

[8] H. Cui , L. Zhang , T. Zhu , R. Liu , and X. Yuan , “Headache as the Sole Clinical Manifestation of Acute Myocardial Infarction: One Case With Cardiac Cephalalgia and Literature Review,” Coronary Artery Disease 35 (2024): 607–613. 38870021 10.1097/MCA.0000000000001394

[9] C. Villalón , D. Centurión , L. Valdivia , P. de Vries , and P. Saxena , “Migraine: Pathophysiology, Pharmacology, Treatment and Future Trends,” Current Vascular Pharmacology 1, no. 1 (2003): 71–84. 15320857 10.2174/1570161033386826

[10] S. A. Hamed , E. A. Hamed , A. M. Ezz Eldin , and N. M. Mahmoud , “Vascular Risk Factors, Endothelial Function, and Carotid Thickness in Patients With Migraine: Relationship to Atherosclerosis,” Journal of Stroke and Cerebrovascular Diseases 19, no. 2 (2010): 92–103. 20189084 10.1016/j.jstrokecerebrovasdis.2009.04.007

